# Structural and Functional Insights into the Delivery Systems of *Bacillus* and Clostridial Binary Toxins

**DOI:** 10.3390/toxins16080330

**Published:** 2024-07-25

**Authors:** Spiridon E. Sevdalis, Kristen M. Varney, Mary E. Cook, Joseph J. Gillespie, Edwin Pozharski, David J. Weber

**Affiliations:** 1 Department of Biochemistry and Molecular Biology, University of Maryland School of Medicine, Baltimore, MD 21201, USA; kvarney@som.umaryland.edu (K.M.V.); mecook@som.umaryland.edu (M.E.C.); epozharskiy@som.umaryland.edu (E.P.); 2 Institute for Bioscience and Biotechnology Research, University of Maryland, Rockville, MD 20850, USA; 3 The Center for Biomolecular Therapeutics, The University of Maryland School of Medicine, Baltimore, MD 21201, USA; 4 Department of Microbiology and Immunology, University of Maryland School of Medicine, Baltimore, MD 21201, USA; jgillespie@som.umaryland.edu

**Keywords:** binary toxins, pore-forming proteins, insecticidal, biotechnology, oligomerization, molecular mechanism

## Abstract

Pathogenic *Bacillus* and clostridial (i.e., *Clostridium* and *Clostridioides*) bacteria express a diverse repertoire of effector proteins to promote disease. This includes production of binary toxins, which enter host epithelial cells and seriously damage the intestinal tracts of insects, animals, and humans. In particular, binary toxins form an AB-type complex composed of a catalytic subunit that is toxic (A) and an oligomeric cell-binding and delivery subunit (B), where upon delivery of A into the cytoplasm of the host cell it catalytically ADP-ribosylates actin and rapidly induces host cell death. In this review, binary toxins expressed by *Bacillus thuringiensis*, *Clostridioides difficile*, and *Clostridium perfringens* will be discussed, with particular focus placed upon the structural elucidations of their respective B subunits and how these findings help to deconvolute how toxic enzyme delivery into target host cells is achieved by these deadly bacteria.

## 1. Introduction

**Figure 1:**
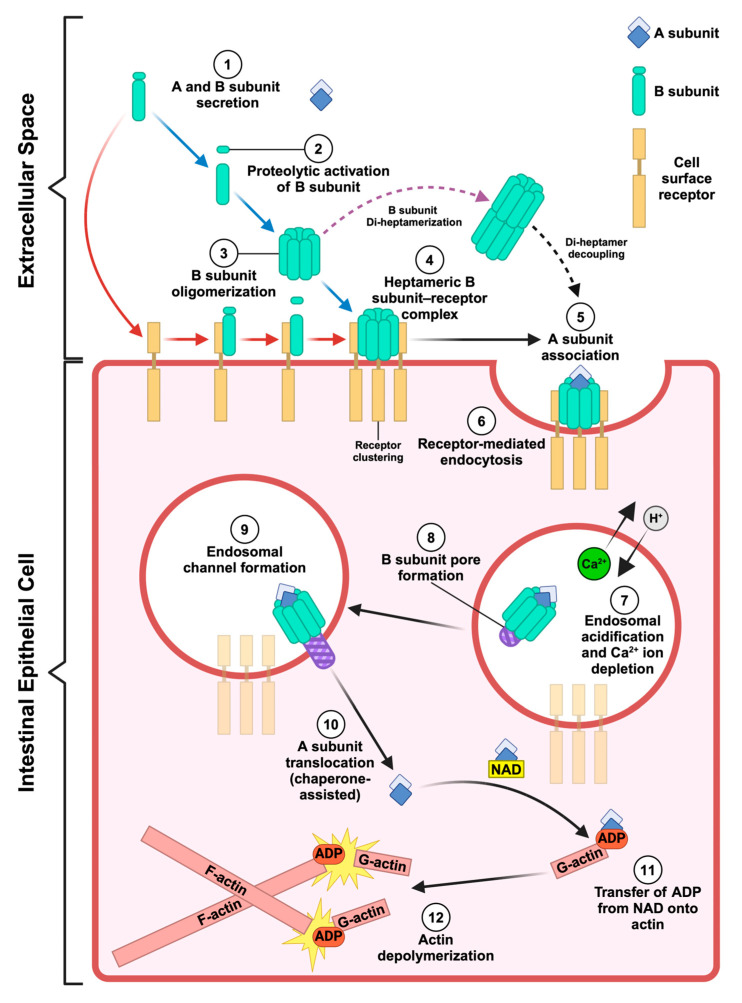
Pathways and states to consider for binary toxin receptor binding, toxin uptake, and cellular toxicity. A and B subunits are secreted independently by vegetative bacteria. One extracellular pathway indicated by blue arrows considers that the B subunit is proteolytically activated and heptamerizes prior to receptor binding [9,10]. Shown by red arrows is another relevant pathway to consider, illustrating that the monomeric B subunit precursor binds to the host cell receptor first, and then it is proteolytically activated, which triggers the clustering of the receptor and facilitates B subunit heptamerization [9,10]. A dashed purple arrow includes a pathway that considers the highly stable CDT di-heptamer structures that were determined at atomic resolution in symmetric and asymmetric states [11]. Likewise, dissociation of di-heptamers into heptamers was found to occur upon decreasing Ca^2+^ ion concentrations associated with receptor-mediated endocytosis [9,10,12,13]. Thus, depletion of Ca^2+^ ions and/or endosomal acidification need to be considered during endosomal delivery of binary toxins, particularly since CDTb-dependent pore formation in the membrane was observed upon lowering Ca^2+^ levels to levels found inside of an endosome [11,12,13]. While not understood at the molecular level, “threading” of the A component through constriction points along the length of the B component “pore” is required for cellular toxicity, and this is proposed to involve host cytosolic chaperones [9,10,14,15]. Once in the cytosol, the A subunit ADP-ribosylates monomeric G-actin, which ultimately leads to host cell death [9,10,11]. This figure was created with the program BioRender.

AB toxins are among the most potent biomolecules produced by pathogenic bacteria. They play a major role in both animal and human disease, with some even having the potential to be used maliciously as biological weapons (e.g., anthrax toxin) [1]. Notably, pathogenic members of the *Bacillus*, *Clostridium*, and *Clostridioides* genera employ AB-type binary toxins to elicit infectious disease. Binary toxins serve as key components of their effector arsenals, targeting the intestinal tracts of insects, animals, and humans [2]. These pore-forming toxins (PFTs) share archetypical similarities, including the following: (1) their A and B subunits are secreted independently of one another, (2) the B subunit binds to cell surface receptors, mediates toxin endocytosis, and facilitates endosomal trafficking of the A subunit into the cytosol, and (3) the A subunit, an ADP-ribosyltransferase (ART), modifies actin through the covalent addition of ADP-ribose acquired from nicotinamide adenine dinucleotide (NAD) at actin position R177, preventing actin filamentation (Figure 1) [1,2,3]. To date, the binary toxin family includes *Clostridioides difficile* transferase (CDT; CDTa and CDTb), *Clostridium perfringens* iota toxin (iota toxin; Ia and Ib) and binary enterotoxin (BEC; BECa and BECb), *Clostridium spiroforme* toxin (CST; CSTa and CSTb), *Clostridium botulinum* C2 toxin (C2; C2I and C2II), and vegetative insecticidal proteins 1 and 2 (Vpb1 and Vpa2) from *Bacillus thuringiensis* [2,4,5]. In addition, anthrax toxin is historically discussed in the context of binary toxin structure and function [2,6]. However, it is important to note that anthrax toxin is technically not a binary toxin as it is an assembly of three independently secreted proteins, which include two enzymatic subunits, lethal factor (LF) and edema factor (EF), and a cell-binding/translocase subunit known as the protective antigen (PA) [7]. The similarities between anthrax toxin and clostridial binary toxins led to speculation that the B component of binary toxins, such as CDTb, bound to three enzymatic partners, an assumption later disproven by cryo-EM studies that revealed CDTa and CDTb form a one-to-one bipartite structure [8]. The structural and functional similarities of the PA to the B subunits of toxins within the binary toxin family make it a necessary and foundational inclusion in the discussion of them. Recent advances are reviewed here, including structures and functional relationships involving bacterial binary and binary-like toxins from pathogenic members of the *Bacillus*, *Clostridium*, and *Clostridioides* genera. How these discoveries reinforced and/or challenged several ongoing molecular mechanisms proposed for inducing toxicity are also discussed. 

## 2. *B. thuringiensis* Toxins

### 2.1. B. thuringiensis

*B. thuringiensis* (*Bt*) is a Gram-positive, aerobic, spore-forming, entomopathogenic soil bacterium found in various ecological habitats [16]. *Bt* is well known for expressing an extensive repertoire of insecticidal toxins. Given this beneficial property, *Bt*-derived toxins have been genetically engineered into crops and used in disease prevention [17]. The most ubiquitously employed of these insecticidal toxins are the three-domain crystal (Cry) and cytolytic proteins (Cyt) [18,19]. The diversity and specificity of Cry and Cyt proteins have allowed them to serve as effective, environmentally friendly agents against pest species of *Lepidoptera* (butterflies and moths), *Diptera* (mosquitoes and other flies), *Coleoptera* (beetles), as well as other harmful arthropods [18,19,20]. These attributes have also made Cry and Cyt proteins useful in targeting vector populations that transmit arbovirus diseases such as chikungunya, dengue, and Zika virus [18,19]. Some Cry proteins also function in a binary toxin-like manner. Notably, this includes the Cry23Aa and Cry37Aa proteins expressed by *Bt* that work in concert to elicit toxicity towards *Cylas puncticollis* (the sweet potato weevil) and *Anthonomus grandis* (the cotton boll weevil) [21,22]. In addition to Cry and Cyt, *Bt* produces several secreted insecticidal proteins (Sips) and vegetative insecticidal proteins (Vips) upon germination [16]. In particular, Vip proteins have emerged as useful alternatives in generating pest-resistant crops and are considered the second-generation of *Bt*-derived insecticidal proteins [16]. These proteins share little sequence and structural homology and bind to different receptors than Cry and Cyt proteins [16,19]. As such, they are used increasingly with the Cry and Cyt proteins expressed in rice, corn, and cotton to enhance protection of these crops from insect damage [16,19]. 

### 2.2. Vegetative Insecticidal Proteins

The application of *Bt*-derived insecticidal proteins was rapidly adopted by farmers across the world after their introduction in the mid-1990s [23]. As of 2020, 82 percent of the total maize acres in the US were sown with *Bt* transgenic maize [4]. However, much like the pervasive use of antibiotics which has led to the emergence of antimicrobial resistance (AMR), so too has extensive genetic modification of crops led to the emergence of *Bt* toxin-resistant pests. Notorious among them is *Diabrotica virgifera virgifera*, the western corn rootworm (WCR), which has acquired resistance to Cry3Bb1 and Gpp34Ab1/Tpp35Ab1 (formally known as Cry34Ab1 and Cry35Ab1) [4,24]. This rise in transgenic crop resistance has motivated the search for new tools to combat crop infestations. These include Vpb1 and Vpa2 (formerly Vip1 and Vip2), which are toxic towards pests of the *Hemiptera* (piercing–sucking True Bugs) and certain *Coleoptera* species [16]. Vpb1 and Vpa2 work in concert as an AB-type binary toxin system, where Vpb1 binds to insectile midgut receptors and forms an endosomal channel used by Vpa2 to enter the cytosol of target cells. Following this translocation, Vpa2 then ADP-ribosylates actin, leading to cytoskeletal disruption and lethal cytotoxicity [16].

**Figure 2:**
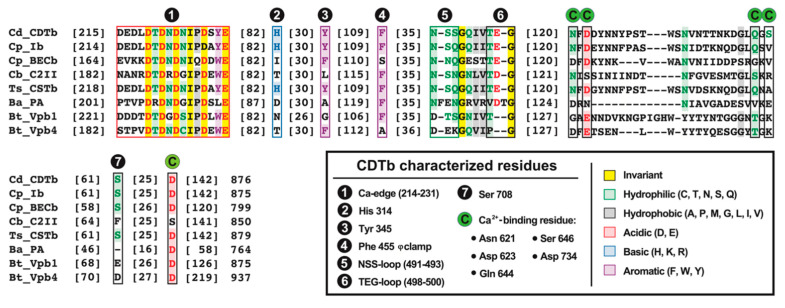
Comparative analysis of B subunits from select clostridial and *Bacillus* binary toxins. Sequences were retrieved from UniProt or NCBI databases and were aligned using MUSCLE (default parameters) with structural and biochemical data superimposed [26]. Cd, *Clostridioides difficile* (CDTb: F5B5U8); Cp, *Clostridium perfringens* (Ib: Q46221, BECb: X5HZK7); Ts, *Thomasclavelia saccharogumia* expression system (CSTb: WP_227053651); Cb, *Clostridium botulinum* (C2II: D4N872); Ba, *Bacillus anthracis* (PA, P13423); and Bt, *Bacillus thuringiensis* (Vpb4: A0A2U5FTM5, Vpb1: M1QYU2). Bracketed values indicate the number of aligned residues between key structural and functional features for the B subunits presented. The numbering scheme above the alignment indicates the order in which these key features appear downstream of the C-terminus. Residue numbering is carried out in respect to CDTb.

Another promising Vip expressed by *Bt* is Vpb4Da2, the first insecticidal Vpb4 protein exhibiting commercial-level control against the WCR [4,24]. Vpb4Da2 is composed of protective antigen 14 (PA14) and Binary-toxB protein family domains [4]. However, unlike the Vpb1/Vpa2 binary toxin system, Vpb4Da2 does not recruit or require an enzymatic partner to facilitate toxicity [4]. This was observed upon feeding WCR larvae Vpb4Da2 alone, which led to the blebbing of midgut epithelial cells six hours post-ingestion, the sloughing off of cellular debris in the WCR lumen, and the partial disruption of the apical microvilli layer 48 h post-ingestion, with nearly complete loss of the microvilli layer 96 h post-ingestion [4]. Fascinatingly, assessment of the amino acid sequence of Vpb4Da2 revealed that an alanine is present in place of the phenylalanine found in other binary toxin B subunits (Figure 2) [25]. As discussed below (Section 5), this phenylalanine is highly conserved and critical for A subunit unfolding and translocation. These observations suggest Vpb4Da2 is unlikely to serve as a transporter of a toxic payload due to the importance this phenylalanine plays in enzymatic A subunit linearization and translocation. This is further reinforced by the ability of Vpb4Da2 to be highly toxic on its own [4]. Despite this, the mode of action for Vpb4Da2 remains unclear. Regardless, Vpb4Da2 shows structural homology to the PA from anthrax toxin and C2II from *B. botulinum* C2 toxin, qualifying its inclusion in this discussion of bacterial pore-forming binary toxins [4]. Furthermore, studies were performed to benchmark the safety of Vpb4Da2, confirming that it is eventually degraded by gastrointestinal proteases in vitro and shows no signs of toxicity in CD-1 mice after oral challenge [25]. These results indicate that Vpb4Da2 (and potentially other Vpb4 toxins) are promising additions to the current repertoire of *Bt*-derived toxins used to protect crops from insect-derived destruction. 

## 3. Structure and Function of the *B. thuringiensis* Binary Toxins

### 3.1. Vpb1/Vpa2

Vpb1/Vpa2 align neatly within the binary toxin paradigm. That is, only when Vpb1 and Vpa2 are both present will toxicity towards the digestive tracts of certain species of *Coleopteran* and *Hemipteran* manifest [17,27]. While the mechanism of action for Vpb1/Vpa2 is not fully established, the process by which cellular intoxication occurs has been partially elucidated by exploring Vpb1 and Vpa2 association and function from various strains of *Bt* and *B. cereus*. Generally, a toxin is secreted by *Bt* in the midgut, where Vpb1/Vpa2 are then proteolytically cleaved by trypsin/trypsin-like proteases in the basic environment (pH 9–11) of insect midgut juices [17,28]. This digestion processes protoxins Vpb1 (100 kDa) and Vpa2 (52 kDa) into mature, active proteins of 80 kDa and 45 kDa size, respectively [17,29]. This proteolysis is critical for Vpb1 oligomerization and Vpa2 translocation [30]. Structural analyses of mature Vpa2 identified two domains, the N-terminal Vpb1-binding domain, and an NAD-binding, ADP-ribosylating C-terminal domain [31]. Unsurprisingly, Vpa2 shares sequence and structural homology with the catalytic domains of CDTa and Ia [30]. In agreement with the binary toxin B subunits expressed by clostridial bacteria, proteolytic cleavage of its N-terminal domain activates Vpb1, promoting heptamerization [28]. This is observed for Vpb1Ac (expressed in *Bt* strain HD201), which forms a circular heptameric assembly after proteolytic processing of its N-terminus [17,28]. The bound Vpb1Ac–Vpa2Ac complex is then endocytosed by brush border membrane vesicles (BBMVs) within the midgut of cotton aphids [17,28,32].

This Vpb1/Vpa2 system is found across many strains of *Bt*, culminating in varying insectile specificities. For example, Vpb1Ad1 and Vpa2Ag1 produced by *Bt* strain HBF-18 (CGMCC 2070) exhibits toxicity against the larvae of *Holotrichia parallela,* a noctuid moth that devastates cash crops across East Asia [27,33]. As expected, in the absence of Vpa2Ag1, histopathology revealed no obvious degradation of *H. parallela* midgut tissue [27]. Alternatively, in the presence of both Vpb1Ad and Vpa2Ag, considerable destruction of the midgut occurred as a product of actin dissociation, culminating in vacuolization of the cytoplasm and microvilli abscission [27]. These findings further reinforce the role Vpb1 serves as a receptor of Vpa2, highlighting this binary toxin relationship and showcasing their potential as tools for protecting crops from insects with increasing resistance to the currently employed *Bt*-derived toxins [27].

### 3.2. Vpb4

**Figure 3:**
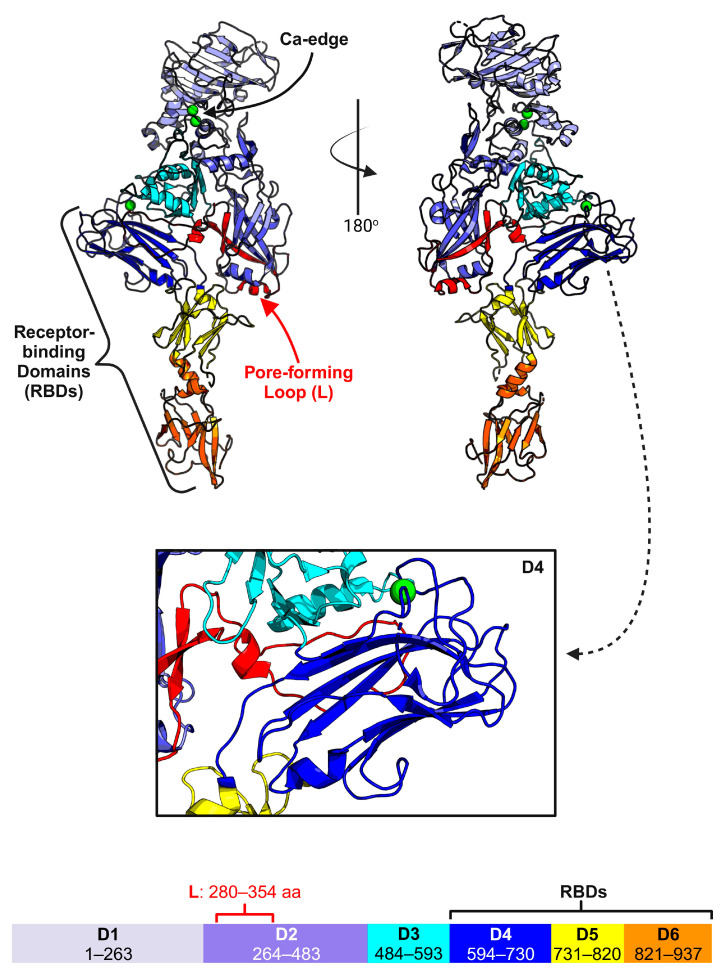
Crystal structure of *Bacillus thuringiensis* Vpb4Da2. This structure identifies some of the key regions of Vpb4Da2 in its “prepore” state that are required for its host cell receptor binding and pore-forming activities. (A) Crystal structure of pro-Vpb4Da2 colored by its domains (D1–D6). The predicted pore-forming loop in the prepore conformation is highlighted in red. The putative receptor-binding domains and the Ca-edge are also indicated. Ca^2+^ ions are represented by green spheres. A zoomed-in view of D4 bound to a Ca^2+^ ion is show in the inset. This structure was modified from PDB: 7MJR [4]. This figure was created with the program BioRender.

Great strides have been made to discern the structure and function of Vpb4. X-ray crystallography was used to resolve the structure of Vpb4Da2 at atomic resolution (3.22 Å), which elucidated six structural domains (Figure 3) [4]. Domain 1 (residues 1–263) encompasses a PA14 domain, contains a ten-stranded β-sandwich conformation similar in structure to bacterial carbohydrate-binding modules, six small α-helices, and features two proximal Ca^2+^-binding sites that are highly conserved among binary toxins [4,34]. The region directly encompassing this dual Ca^2+^ ion site is referred to as the “Ca-edge” in anthrax toxin PA and binary toxin B subunits and is situated at the N-terminus of active Vpb4Da2 (Figure 2) [14,15,34]. Domain 1 also contains the trypsin cleavage site necessary for Vpb4Da2 oligomerization, as revealed by fluorescence-based SDS-PAGE analyses of Vpb4Da2 and Vpb4Da2 variants [4]. Domain 2 (residues 264–483) contains a β-hairpin motif, seven anti-parallel β-strands, and an additional three α-helices [4]. A long stem region/pore-forming loop (residues 280–354) is present in Domain 2, which is thought to undergo a conformational change in an acidic pH to form a β-barrel pore. Domain 3 (residues 484–593) contains a seven-stranded β-sandwich and four α-helices [4]. Domain 4 (residues 594–730) contains a β-jelly roll fold and a Ca^2+^-binding site [4]. Interestingly, Domain 4 resembles the receptor-binding domain 1 (RBD1) from CDTb, which also contains a Ca^2+^-binding site and whose β-sheet-rich structure is intrinsically dependent on the presence of Ca^2+^ ions [11,12]. Domain 5 (residues 731–820) has six β-strands oriented in an immunoglobulin-like fold and one α-helix, whereas Domain 6 (residues 821–937) has seven β-strands oriented in an immunoglobulin-like fold and two α-helices [4].

Using the knowledge that domain 4 from the PA of anthrax toxin, the receptor binding domains (residues 616–876) in CDTb, and Domain 4 (residues 616–875) in Ib are involved in receptor binding, domains 4–6 of Vpb4Da2 were assessed for receptor recognition [4,11,14]. To verify this, the authors conducted a carboxyl terminal domain swap analysis using Vpb4C.6693, a nonlethal Vpb4 with 77% protein sequence homology to Vpb4Da2, generating two Vpb4 chimeras, Vpb4Da2-Vpb4C.6693 (chimera-1) and Vpb4C.6693-Vpb4Da2 (chimera-2) [4]. Chimera-1 was found to be non-larvicidal against WCR. Alternatively, Chimera-2, containing domains 1–3 of Vpb4C.6693 and domains 4–6 of Vpb4Da2, displayed WCR larval susceptibility on par with wild-type Vpb4Da2 [4]. These results show the significance of domains 4–6 in Vpb4Da2 toxicity and given the structural homology to binary toxin receptor-binding domains; these observations suggest Vpb4Da2 receptor binding is conferred by unknown solvent-exposed residues within these domains [4]. Ultimately, these findings present a major development in understanding the function of Vpb4 and other *Bt*-expressed insecticidal binary toxins. To continue this progress, the structure of heptameric Vpb4Da2 and the receptor it binds to must be examined further. Such research is needed to decipher the molecular mechanism of Vpb4 toxicity, as this is critically important for its safe and effective commercial use. 

## 4. Clostridial Binary Toxins

### 4.1. C. perfringens

*C. perfringens* is an anaerobic, Gram-positive, nonmotile, subterminal, spore-forming rod expressing an extensive arsenal of PFTs across different strains [35]. *C. perfringens* is a major cause of human foodborne disease and is one of the most common instigators of food poisoning in the United States of America (USA) [35]. Contaminated poultry, meat, and meat-based products serve as the main source of infection for humans, resulting in nearly a million cases of food-related illness in the USA annually [35]. In humans, illness is typically short-term (12–24 h) and includes symptomology such as watery diarrhea and abdominal pain [35]. Analogous to *C. difficile*, interventions utilizing broad-spectrum antibiotics can lead to *C. perfringens* infection as a consequence of gut microbiome disruption [35]. Furthermore, depending on the strain of *C. perfringens*, severe symptoms can manifest, including intestinal wound infection, septicemia, enterotoxaemia, and enteritis [35].

There are a large number of varying PFT toxins produced by *C*. *perfringens*. In the case of *C. perfringens* type B and D, the PFT known as epsilon toxin (Ext) causes enterotoxaemia in domestic ruminants by entering the blood stream through the intestinal wall and preferentially accumulating in brain and kidney tissue [36]. *C. perfringens* type C produces *C. perfringens* beta toxin (CPB), a potent hemolysin-like β-PFT that causes intestinal damage, and lethal necrotic enteritis in both animals and humans through the targeting of endothelial cells [36,37]. Other β-PFTs produced by *C. perfringens* strains are specific to livestock, including *C. perfringens* type F Enterotoxin (CPE) [38]. CPE oligomerizes after binding to cell surface receptors termed claudins, leading to the formation of cytotoxic β-pores that disrupt gastrointestinal homeostasis in mammals [38]. Another hemolysin-like β-PFT produced by *C. perfringens* type G is necrotic enteritis B-like toxin (NetB), which causes necrotic enteritis in chickens [39,40]. In addition to these toxins, *C. perfringens* expresses two binary toxins, the iota toxin, which is expressed by *C. perfringens* type E, and the binary enterotoxin of *C. perfringens* (BEC) expressed in *C. perfringens* strains OS1 and TS1 [41,42]. For our purposes, this review focuses only on the iota toxin expressed by *C*. *perfringens* type E. 

Overfeeding, stress, and cold conditions can trigger *C. perfringens* type E overgrowth in bovines, promoting the expression of iota toxin [41]. Iota toxin consists of two non-linked subunits: Ia, the catalytic subunit, and Ib, the translocase/pore-forming subunit that forms a heptameric assembly of Ib monomers [14,41]. Once Ia enters the cytosol of intestinal mucosal cells, it ADP-ribosylates G-actin and prevents the polymerization of F-actin, leading to cytoskeletal destruction, intestinal epithelial cell permeability, and cell death [15,41,43]. Fascinatingly, Ia (as well as CDTa and C2I) was shown to have ART activity against Arp2 of the Arp2/3 complex at R179, suggesting these toxins use a multifaceted approach to inhibit actin polymerization [44]. Iota toxin expression was also found to induce antibiotic-associated enterotoxaemia in rabbits and hemorrhagic enterocolitis and sudden death in calves and lambs [43]. This sudden induction of iota toxin expression and the havoc it can wreak on livestock make it an important virulence factor to study with regard to *C. perfringens* pathogenesis.

### 4.2. Clostridioides Difficile

Parallel to *C. perfringens*, *C. difficile* is an anaerobic, Gram-positive, spore- and toxin-producing bacillus [45]. Its sporulating function makes *C. difficile* persistent and oftentimes difficult to eradicate even when using harsh cleaning methods [8,46]. *C. difficile* infection (CDI) is prevalent in nosocomial settings, with 500,000 estimated cases and 29,000 deaths reported per year in the USA [45]. Clinical outcomes of CDI include severe diarrhea and damage to the colonic epithelium, resulting in pseudomembranous colitis. CDT is also associated with increased rates of mortality for immunocompromised and elderly individuals [8,46]. Typically, a diverse and robust microbiome protects against the activation and colonization of *C. difficile* in the large intestine of healthy individuals [47]. It is upon disruption of those protective commensal bacteria that *C. difficile* is able to flourish. Factors that promote this disruption include different chemotherapies and antibiotic interventions. 

*C. difficile* has an extensive AMR repertoire, including resistance to lincomycin, clindamycin, aminoglycosides, tetracyclines, macrolides, cephalosporins, penicillin, and fluoroquinolones [46]. As such, it is difficult to discuss *C. difficile* without addressing its propensity for acquiring AMR. The two are interwoven to such a degree that initially CDI was referred to as “clindamycin-associated colitis” due to the high risk clindamycin posed in inducing CDI [46]. The current antibiotic regimen employed to treat CDI includes vancomycin and fidaxomicin, or metronidazole if the prior two are unavailable [46]. Once *C. difficile* establishes its niche within the gut, disease manifests as a product of toxin secretion, resulting in cytotoxicity, cellular detachment from the intestinal epithelium, cell death, and inflammation of the infection site [45]. These toxins include two large Rho-glycosylating enterotoxins referred to as toxin A (TcdA) and toxin B (TcdB), and a third β-pore-forming binary toxin known as CDT [11,46]. CDT is associated with hypervirulent strains of *C. difficile*, such as the fluoroquinolone resistant ribotype 027 (RT027), resulting in higher frequencies of severe disease and recurrence in patients with CDT-positive strains of *C. difficile* [45,46]. The binary toxin, CDT, is composed of the ADT ribosyltransferase (ART) toxic component, CDTa, and the oligomeric translocase/pore-forming component, CDTb [11,15]. CDT shares high sequence and structural homology with the iota toxin, with CDTa and CDTb showing an 81% and 84% amino acid sequence identity to Ia and Ib, respectively [12]. This similarity between CDT and iota toxin is further highlighted by the interchangeability of their A and B subunits without abolishing or altering toxicity [12]. Therefore, the structure and function of CDT and the iota toxin, with focus placed upon their B subunits, will be discussed in conjunction in the following section.

## 5. Structure and Function of Clostridial Binary Toxins

### 5.1. CDTb and Ib Oligomerization

**Figure 4:**
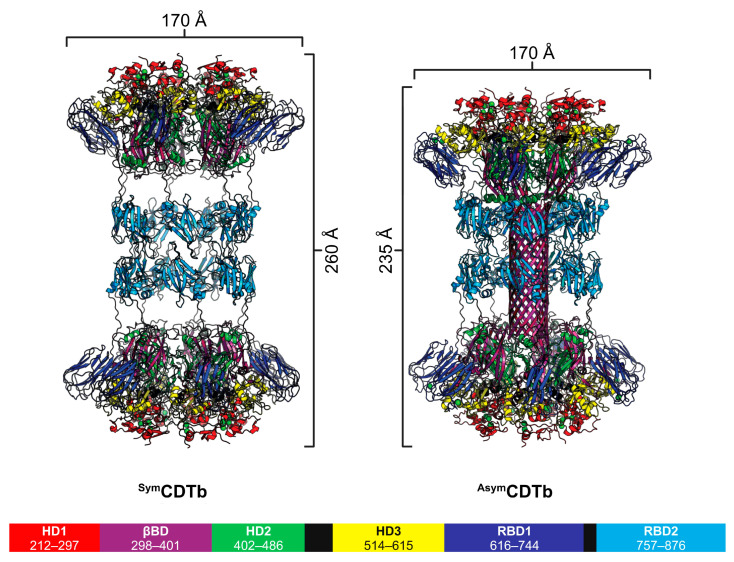
Atomic-resolution structures of di-heptameric CDTb from *C. difficle*. The 3D structures of the CDTb di-heptamers are illustrated in ^Sym^CDTb and ^Asym^CDTb conformations with domains colored and labeled. Structures are modified from PDB: 6UWT (^Sym^CDTb) and 6UWR (^Asym^CDTb) [11]. The di-heptamer structures are highly stable, and can dissociate into “active” heptameric structures upon dissociation of Ca^2+^ from a newly described Ca^2+^-site within its RBD1 domain (residues 616–744). This figure was created with the program BioRender.

Oligomerization of CDTb and Ib occurs under certain conditions upon proteolytic cleavage of pro-CDTb (99 kDa) and pro-Ib (100 kDa) by trypsin or chymotrypsin at their respective N-termini [11,12,43]. During CDTb proteolysis, the signaling peptide (SD; residues 1–43) and the activation domain (AD; residues 44–211) are removed, producing active CDTb (75 kDa) that consists of residues 212–876 [11,12], whereas the active form of Ib (80 kDa), after removal of its SD and AD, consists of residues 216–875 [14,41]. Interestingly, sizing studies of active CDTb performed via sedimentation velocity analytical ultracentrifugation (AUC) and size-exclusion chromatography/multiangle light scattering (SEC-MALS) to determine the stoichiometry of CDTb oligomerization found monomeric CDTb to be the predominant species present in vitro (95 ± 2%), with a novel di-heptameric macromolecular assembly (1.0 MDa) detected at lower levels (<4–6%) [11]. Subsequent addition of CDTa shifted the equilibrium towards the di-heptameric species (>25%). This was followed by single-particle cryoelectronic microscopy (cryo-EM) studies showing that, in the absence of CDTa and under soluble conditions with millimolar Ca^2+^ ion concentrations, the only higher molecular weight species observed for activated CDTb were the di-heptameric oligomerization states [11]. They included two unique structures: (1) Asymmetric CDTb (^Asym^CDTb), where one of the CDTb heptamers has an extended β-barrel domain (βBD; residues 298–401) while the other is in the prepore state, and (2) symmetric CDTb (^Sym^CDTb), where both CDTb heptamers are in the prepore state (Figure 4) [11]. These findings are a deviation of the heptameric or octameric (in respect to anthrax toxin PA) assemblies that binary toxin B subunits typically form. It is hypothesized that these di-heptamers represent a mechanism employed by CDTb to protect its central core against proteolytic cleavage by extracellular enzymes and/or evade host immune responses [11,12,13]. Regardless of whether CDTb is in the asymmetric or symmetric conformation, both share a unique central, donut-shaped assembly consisting of an interface between the receptor-binding domain 2 (RBD2) domains of one heptamer with the RBD2 domains of the other [8,11,15]. Despite the presence of an RBD2 analogue, this donut-shaped assembly has yet to be observed in the iota toxin.

### 5.2. CDTb/Ib Heptamerization and Receptor Binding

Given the sequence and structural similarities between CDTb and Ib, it follows that they share the lipolysis-stimulated lipoprotein receptor (LSR), a type-I single-pass transmembrane protein, characterized by an extracellular immunoglobulin-like domain and an extended intracellular segment, as their primary host cell surface receptor [43,48,49]. Clostridial binary toxins also bind to CD44, a type-I cell surface glycoprotein typically associated with lipid rafts (cholesterol-rich regions of the cell membrane), to intoxicate cells [50]. For example, it was observed that the Ib heptamer associates with lipid rafts and that CD44 was found occupying lipid rafts in Ib-treated Vero cells [51,52,53]. This toxin–host interface then triggers cellular uptake of the toxin via endocytosis. However, despite the surge in structural information for both CDT and iota toxin, the mechanisms by which endocytosis occurs for these toxins remain somewhat ambiguous. For example, there is uncertainty about whether proteolytic activation occurs prior to or after B subunit binding to the cell surface receptor. This uncertainty has fueled discourse on whether the B subunit binds to the cell as an inactivated monomer or as a heptamer (Figure 1) [10]. Moreover, studies indicate that LSR is not a component of lipid rifts and will instead localize to them after associating with CDTb [54]. Interestingly, experiments exploring the association between CDTb and LSR revealed CDTb, in the absence of CDTa, promotes cytotoxic effects such as cell rounding in cells expressing LSR [55]. Specifically, this is a consequence of pore formation, as the receptor-binding domains of CDTb alone were not sufficient in promoting cell rounding [55]. Furthermore, for HeLa cells that did not express LSR and for LSR knock-out CaCo-2 cells, CDTb-induced cell rounding was impeded [55]. Likewise, it has been observed that upon LSR association and heptamerization, Ib forms cytotoxic ion-permeable channels which facilitate the release of K^+^ and Na^+^ influx in Vero cells [43,56,57,58]. Nevertheless, the formation of the heptamer, whether prior to or after coming into contact with LSR, is pivotal for CDT and iota toxin function. As such, efforts have been made to inhibit their oligomerization. Most notably, these efforts include the development of monoclonal antibodies (e.g., BINTOXB/9 and BINTOXB/22) which inhibit trypsin-induced CDTb oligomerization and subsequently CDT toxicity [59].

### 5.3. Toxin Uptake and CDTa/Ia Translocation

**Figure 5:**
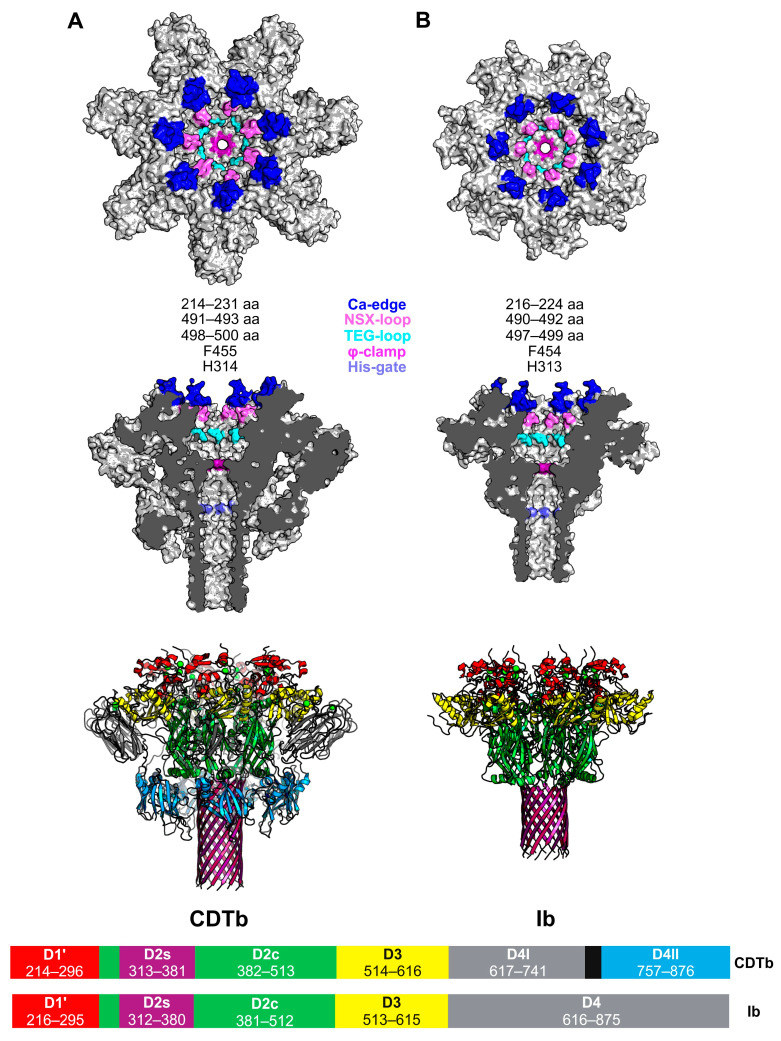
Atomic model of constriction sites and domain structures for activated *C. difficle* CDTb and *C. perfringens* Ib. These structures are illustrated to show the key regions of active CDTb and Ib required for host cell receptor binding, pore-forming activities, and catalytic subunit translocation. (**A**) The four constriction sites and the φ-clamp of the CDTb-pore: Ca-edge (214–231 aa), NSS-loop (491–493 aa), TEG-loop (498–500 aa), φ-clamp (F455), and His-gate (H314). Structure modified from PDB: 7VNN [15]. (**B**) The four constriction sites and the φ-clamp of the Ib-pore: Ca-edge (216–224 aa), NSQ-loop (490–492 aa), TEG-loop (497–499 aa), φ-clamp (F454), and His-gate (H313). Structure modified from PDB: 6KLX [14]. Domain structures and their corresponding domain maps are presented below the atomic models of Ib and CDTb. This figure was created with the program BioRender.

Following oligomerization and receptor binding, the N-terminal domain of CDTa (residues 1–217) associates with heptamerization domain I (HD1; residues 212–297) of heptameric CDTb [11,12,15]. Similarly, the N-terminal domain of Ia (residues 1–210) associates with domain 1′ (D1′; residues 216–295) of oligomerized Ib [14]. The Ca-edge (CDTb; residues 214–231, Ib; 216–224) is the first constriction point encountered by CDTa/Ia upon B subunit association (Figure 5) [14,15]. This association between toxin and receptor is a major driving force for the endocytic uptake of CDT and iota toxin [49]. For iota toxin specifically, dynamin was also determined to be required for its endocytic uptake [9,60]. The second constriction point, a short loop consisting of an arginine and two serine residues in CDTb (NSS-loop; residues 491–493), is also significant in CDTa binding (Figure 5A) [8,15]. In Ib, this loop is composed of an arginine, a serine, and a glutamine residue (NSQ-loop; residues 490–492) (Figure 5B) [14]. This “NSX-loop” also appears in CSTb (as NSQ) and C2II (as NSN). This is distinct, but akin to the function of the α-clamp located in the funnel rim of the anthrax toxin PA, which binds to helical portions of LF and aids in LF translocation through the PA pore [8,61]. In both CDTb and Ib, the third and final constriction site prior to the β-barrel is the “TEG-loop” (Figure 5), which is conserved across clostridial binary toxins (Figure 2) [14,15].

### 5.4. Mechanisms of Endosomal Export for CDTa/Ia

A critical regulatory feature in translocation for CDTa/Ia is the φ-gate or φ-clamp, which is positioned in a ring at the entrance of the pore created by CDTb/Ib (Figure 2 and Figure 5) [8,14,15]. In CDTb, the φ-clamp consists of a phenylalanine (F455) from heptamerization domain 2 (HD2), contributed by each CDTb protomer [8,11,15]. In Ib, this phenylalanine (F454) is donated by each Ib protomer from the D2c domain [14]. As the β-barrel extension is triggered, the φ-clamp enters a conformational constricted “closed” state [11,12]. In CDTb, the closed φ-clamp of the extended β-barrel forms a passageway with a diameter of 3 Å, whereas the non–β-barrel, or “open” state pore diameter is 12.5 Å [11]. A similar narrowing is observed for Ib with a closed φ-clamp diameter of 6 Å; however, structural data of prepore Ib are lacking [14]. The putative mechanism suggests that the acidic environment of mature endosomes facilitates the N-terminal threading of CDTa/Ia through the φ-clamp followed by extended-chain Brownian ratchet model-mediated translocation through the β-barrel [14,15]. Aided by pH-mediated ‘flipping’ of NSX-loop conformations, the φ-clamp is a critical constriction point required for the unfolding and passage of the enzymatic component through the β-barrel [14,15]. The fifth and final constriction occurs at residue H314 (CDTb) and H313 (Ib), located in the β-barrel stem just below the φ-clamp (Figure 5) [14,15]. Upon channeling of the enzymatic subunit through the β-barrel stem, chaperones such as heat-shock proteins 70 (Hsp70) and 90 (Hsp90), and peptidyl-prolyl cis-/trans-isomerases (PPIases) such as cyclophilin A, are proposed to then aid CDTa/Ia in entering the cytosol [49,62,63,64,65]. The current model suggests these host cell factors facilitate the transmembrane transport of the enzymatic payload through interactions with the ADP-ribosyltransferase domain of the enzyme [9,66]. Furthermore, this chaperone intervention is necessary as the enzymatic A subunits, after having passed through the sterically constrictive B subunits, will be at least partially unfolded and require refolding [63]. Moreover, inhibition of these chaperones and PPIases has been performed and the results from these studies have presented a novel mechanism of action to consider as well as a new pharmacological strategy against CDT-producing strains of *C. difficile* [9,65,67]. 

As was discussed, CDTb and Ib endosomal pore formation is partially dependent upon endosomal acidification [8,43,68]. Interestingly, this acidification is accompanied by the transport of Ca^2+^ ions out of the endosomal space and into the cytosol via Ca^2+^-permeable channels, resulting in a significant drop in free Ca^2+^ ion concentration after endosome formation [69,70]. While the importance of the constriction motifs discussed and the acidic pH of mature endosomes in the function of CDTb/Ib cannot be understated, an additional factor impacting toxin translocation centered upon Ca^2+^ ion concentration has emerged and is deserving of exploration. 

## 6. Shifting the Paradigm and Conclusions

As alluded to above for Vbp4Da2, RBD1 of CDTb also contains a Ca^2+^ ion binding site that is hypothesized to play a role in CDT toxicity [11,12]. It was the crystal structure (PDB: 6UWI) of CDTb that first indicated that RBD1 coordinates a Ca^2+^ ion [11]. The presence of this Ca^2+^ ion was confirmed by inductively coupled plasma mass spectrometry (ICP-MS) [11]. Nuclear magnetic resonance (NMR) of RBD1 then followed as a means to further probe RBD1 Ca^2+^-binding [11]. Two-dimensional [^1^H, ^15^N]-heteronuclear single quantum coherence spectroscopy (2D [^1^H, ^15^N]-HSQC) data for apo-RBD1 displayed severe line-broadening effects and a lack of residue dispersion consistent with a protein in a state undergoing conformational exchange in the millisecond to microsecond timescale [11]. In the presence of 6 mM Ca^2+^ (approximately three times the concentration found in the extracellular space), the 2D [^1^H, ^15^N]-HSQC spectral line-width values narrowed and significant residual dispersion congruent with a structured, folded protein emerged [11]. This relationship with Ca^2+^ is intriguing as it brings into question the biological role that such a dramatic Ca^2+^-induced conformational change plays in CDT toxicity. 

Structural and functional studies probing CDTb pore formation have revealed that the unfolded state of RBD1 is essential for β-barrel extension [13]. Specifically, atomic resolution cryo-EM structures of Ca^2+^-depleted CDTb and a Ca^2+^-binding mutant of CDTb (CDTb^D623A/D734A^) exhibited extended β-barrel structures [13]. Furthermore, surface plasmon resonance (SPR) and electrochemical impedance spectroscopy (EIS) demonstrated how both Ca^2+^-depleted CDTb and CDTb^D623A/D734A^ were able to associate with a phospholipid biochip, while CDTb in the presence of Ca^2+^ could not [13]. Together, these findings suggest that part of the endosomal pore-forming function of CDTb is driven by the Ca^2+^-depleted environment of the mature endosome.

**Figure 6:**
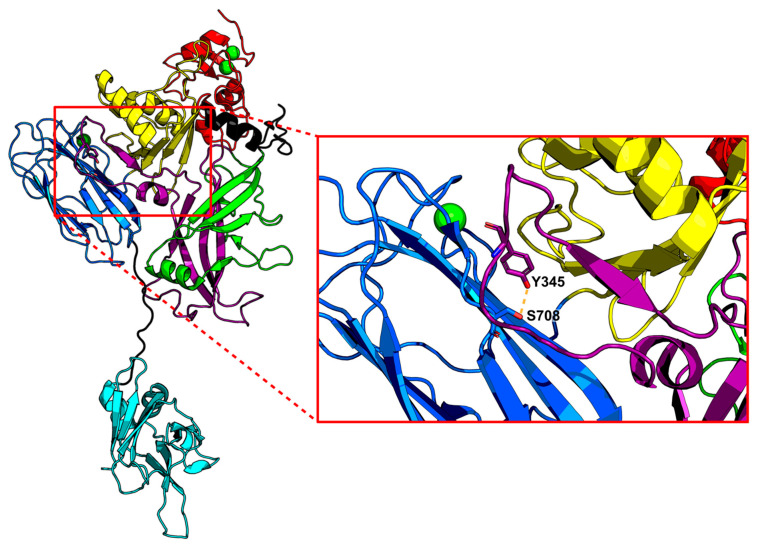
The interface of the βBD (violet; residues 298–401) and Ca^2+^-bound RBD1 (blue; residues 616–744) shown for a prepore CDTb protomer. Close-up view of the hydrogen bond interaction (yellow dotted line) between Y345 of βBD and S708 of RBD1 is highlighted in the inset. Domains are colored according to Figure 4. Structure modified from PDB: 6UWI (^Sym^CDTb). For CDTb, the dissociation of Ca^2+^ from this novel RBD1 site can be considered in other binary toxins for A subunit delivery via endosomes, in addition to the lowering of pH, as is typically required for most binary toxins (i.e., anthrax and others). This figure was created with the program BioRender.

Further, the structure of CDTb revealed that in the prepore conformation, RBD1 and the βBD interface with one another through a hydrogen bond between S708 from RBD1 and Y345 from the βBD (Figure 2 and Figure 6). A disruption of this H-bond may occur as RBD1 unfolds in the absence of Ca^2+^, allowing the βBD to unfurl and assemble the β-barrel channel. This potential sensitivity to Ca^2+^ may be present in other binary toxins. The Ca^2+^-binding site residues (N621, D623, Q644, S646, and D734) in CDTb are highly conserved among Ib and CSTb, with the exception of S646, which appears as a valine for Ib and an alanine for CSTb (Figure 2). Nevertheless, there is a serine directly downstream of the valine in Ib, and an arginine present directly downstream of the alanine in CSTb, both of which contain oxygen atoms that could be implicated in coordinating Ca^2+^. These Ca^2+^-coordinating residues are not as well conserved in C2II, BECb, PA, Vpb1, and Vpb4 (Figure 2). Despite this deviation from CDTb, many of these alternative residues contain the propensity to coordinate Ca^2+^ with three of the five Ca^2+^-coordinating residues found in CDTb RBD1 having conserved substitutions in other binary toxin B subunits (Figure 2). The relevance of this Ca^2+^ binding in CDTb, Ib, and CSTb is reinforced by the conservation of prepore-stabilizing residues S708 and Y345 (Figure 2).

Sequence similarities and recent data outlining the preference of CDTb to extend its β-barrel in the absence of Ca^2+^ ions indicate that the role of Ca^2+^ may have biological relevance in promoting toxicity across diverse clostridial binary toxins. Additionally, binary toxins expressed by *Bacillus* species may also contain a Ca^2+^-dependence that has been overlooked. As mentioned earlier, Vpb4Da2 has a Ca^2+^ ion bound to its domain 4, a domain that is structurally similar to RBD1 from CDTb, suggesting a potential biologically relevant role for toxin Ca^2+^ association/dissociation extending across the *Clostridium*, *Clostridioides*, and *Bacillus* genera. Further investigation into the binary toxin delivery system, especially with relation to Ca^2+^, is therefore paramount to understanding how these binary toxins are delivered into the host cell. 

## References

[1] Jia J., Braune-Yan M., Lietz S., Wahba M., Pulliainen A.T., Barth H., Ernst K. (2023). Domperidone Inhibits Clostridium Botulinum C2 Toxin and Bordetella Pertussis Toxin. Toxins.

[2] Stiles B.G., Pradhan K., Fleming J.M., Samy R.P., Barth H., Popoff M.R. (2014). Clostridium and bacillus binary enterotoxins: Bad for the bowels, and eukaryotic being. Toxins.

[3] Stieglitz F., Gerhard R., Pich A. (2021). The binary toxin of *Clostridioides difficile* alters the proteome and phosphoproteome of HEp-2 cells. Front. Microbiol..

[4] Kouadio J.L., Zheng M., Aikins M., Duda D., Duff S., Chen D., Zhang J., Milligan J., Taylor C., Mamanella P. (2021). Structural and functional insights into the first Bacillus thuringiensis vegetative insecticidal protein of the Vpb4 fold, active against western corn rootworm. PLoS ONE.

[5] Sauka D.H., Peralta C., Pérez M.P., Molla A., Fernandez-Göbel T., Ocampo F., Palma L. (2023). *Bacillus thuringiensis* Bt_UNVM-84, a Novel Strain Showing Insecticidal Activity against *Anthonomus grandis* Boheman (Coleoptera: Curculionidae). Toxins.

[6] Popoff M.R. (2014). Clostridial pore-forming toxins: Powerful virulence factors. Anaerobe.

[7] Michelman-Ribeiro A., Rubinson K.A., Silin V., Kasianowicz J.J. (2021). Solution Structures of *Bacillus anthracis* Protective Antigen Proteins Using Small Angle Neutron Scattering and Protective Antigen 63 Ion Channel Formation Kinetics. Toxins.

[8] Sheedlo M.J., Anderson D.M., Thomas A.K., Lacy D.B. (2020). Structural elucidation of the *Clostridioides difficile* transferase toxin reveals a single-site binding mode for the enzyme. Proc. Natl. Acad. Sci. USA.

[9] Papatheodorou P., Minton N.P., Aktories K., Barth H. (2024). An Updated View on the Cellular Uptake and Mode-of-Action of *Clostridioides difficile* Toxins. Adv. Exp. Med. Biol..

[10] Papatheodorou P., Aktories K. (2017). Receptor-Binding and Uptake of Binary Actin-ADP-Ribosylating Toxins. Curr. Top. Microbiol. Immunol..

[11] Xu X., Godoy-Ruiz R., Adipietro K.A., Peralta C., Ben-Hail D., Varney K.M., Cook M.E., Roth B.M., Wilder P.T., Cleveland T. (2020). Structure of the cell-binding component of the *Clostridium difficile* binary toxin reveals a di-heptamer macromolecular assembly. Proc. Natl. Acad. Sci. USA.

[12] Abeyawardhane D.L., Godoy-Ruiz R., Adipietro K.A., Varney K.M., Rustandi R.R., Pozharski E., Weber D.J. (2021). The importance of therapeutically targeting the binary toxin from *Clostridioides difficile*. Int. J. Mol. Sci..

[13] Abeyawardhane D.L., Sevdalis S.E., Adipietro K.A., Godoy-Ruiz R., Varney K.M., Nawaz I.F., Spittel A.X., Rustandi R.R., Silin V.I., des Georges A. (2023). Membrane binding and pore formation is Ca. bioRxiv.

[14] Yamada T., Yoshida T., Kawamoto A., Mitsuoka K., Iwasaki K., Tsuge H. (2020). Cryo-EM structures reveal translocational unfolding in the clostridial binary iota toxin complex. Nat. Struct. Mol. Biol..

[15] Kawamoto A., Yamada T., Yoshida T., Sato Y., Kato T., Tsuge H. (2022). Cryo-EM structures of the translocational binary toxin complex CDTa-bound CDTb-pore from *Clostridioides difficile*. Nat. Commun..

[16] Gupta M., Kumar H., Kaur S. (2021). Vegetative insecticidal protein (Vip): A potential contender from Bacillus thuringiensis for efficient management of various detrimental agricultural pests. Front. Microbiol..

[17] Syed T., Askari M., Meng Z., Li Y., Abid M.A., Wei Y., Guo S., Liang C., Zhang R. (2021). Correction: Syed, T.; et al. Current Insights on Vegetative Insecticidal Proteins (Vip) as Next Generation Pest Killers. *Toxins* 2020, *12*, 522. Toxins.

[18] Tetreau G., Sawaya M.R., De Zitter E., Andreeva E.A., Banneville A.S., Schibrowsky N.A., Coquelle N., Brewster A.S., Grünbein M.L., Kovacs G.N. (2022). De novo determination of mosquitocidal Cry11Aa and Cry11Ba structures from naturally-occurring nanocrystals. Nat. Commun..

[19] Lai L., Villanueva M., Muruzabal-Galarza A., Fernández A.B., Unzue A., Toledo-Arana A., Caballero P., Caballero C.J. (2023). *Bacillus thuringiensis* Cyt proteins as enablers of activity of Cry and Tpp toxins against Aedes albopictus. Toxins.

[20] Liu L., Li Z., Luo X., Zhang X., Chou S.H., Wang J., He J. (2021). Which Is Stronger? A Continuing Battle Between Cry Toxins and Insects. Front. Microbiol..

[21] Ribeiro T.P., Martins-de-Sa D., Macedo L.L.P., Lourenço-Tessutti I.T., Ruffo G.C., Sousa J.P.A., Rósario Santana J.M.D., Oliveira-Neto O.B., Moura S.M., Silva M.C.M. (2024). Cotton plants overexpressing the Bacillus thuringiensis Cry23Aa and Cry37Aa binary-like toxins exhibit high resistance to the cotton boll weevil (*Anthonomus grandis*). Plant Sci..

[22] Hernández-Martínez P., Khorramnejad A., Prentice K., Andrés-Garrido A., Vera-Velasco N.M., Smagghe G., Escriche B. (2020). The independent biological activity of *Bacillus thuringiensis* Cry23Aa protein against Cylas puncticollis. Front. Microbiol..

[23] Brookes G., Barfoot P. (2020). Environmental impacts of genetically modified (GM) crop use 1996–2018: Impacts on pesticide use and carbon emissions. GM Crops Food.

[24] Yin Y., Flasinski S., Moar W., Bowen D., Chay C., Milligan J., Kouadio J.L., Pan A., Werner B., Buckman K. (2020). A new Bacillus thuringiensis protein for Western corn rootworm control. PLoS ONE.

[25] Edrington T., Wang R., McKinnon L., Kessenich C., Hodge-Bell K., Li W., Tan J., Brown G., Wang C., Li B. (2022). Food and feed safety of the Bacillus thuringiensis derived protein Vpb4Da2, a novel protein for control of western corn rootworm. PLoS ONE.

[26] Edgar R.C. (2004). MUSCLE: A multiple sequence alignment method with reduced time and space complexity. BMC Bioinform..

[27] Geng J., Jiang J., Shu C., Wang Z., Song F., Geng L., Duan J., Zhang J. (2019). *Bacillus thuringiensis* Vip1 functions as a receptor of Vip2 toxin for binary insecticidal activity against Holotrichia parallela. Toxins.

[28] Leuber M., Orlik F., Schiffler B., Sickmann A., Benz R. (2006). Vegetative insecticidal protein (Vip1Ac) of Bacillus thuringiensis HD201: Evidence for oligomer and channel formation. Biochemistry.

[29] Bi Y., Zhang Y., Shu C., Crickmore N., Wang Q., Du L., Song F., Zhang J. (2015). Genomic sequencing identifies novel Bacillus thuringiensis Vip1/Vip2 binary and Cry8 toxins that have high toxicity to Scarabaeoidea larvae. Appl. Microbiol. Biotechnol..

[30] Palma L., Muñoz D., Berry C., Murillo J., Caballero P. (2014). Bacillus thuringiensis toxins: An overview of their biocidal activity. Toxins.

[31] Jucovic M., Walters F.S., Warren G.W., Palekar N.V., Chen J.S. (2008). From enzyme to zymogen: Engineering Vip2, an ADP-ribosyltransferase from Bacillus cereus, for conditional toxicity. Protein Eng. Des. Sel..

[32] Sattar S., Maiti M.K. (2011). Molecular characterization of a novel vegetative insecticidal protein from Bacillus thuringiensis effective against sap-sucking insect pest. J. Microbiol. Biotechnol..

[33] Li E., Qin J., Feng H., Li J., Li X., Nyamwasa I., Cao Y., Ruan W., Li K., Yin J. (2021). Immune-related genes of the larval Holotrichia parallela in response to entomopathogenic nematodes Heterorhabditis beicherriana LF. BMC Genom..

[34] Rigden D.J., Mello L.V., Galperin M.Y. (2004). The PA14 domain, a conserved all-beta domain in bacterial toxins, enzymes, adhesins and signaling molecules. Trends Biochem. Sci..

[35] Mora Z.V., Macías-Rodríguez M.E., Arratia-Quijada J., Gonzalez-Torres Y.S., Nuño K., Villarruel-López A. (2020). *Clostridium perfringens* as foodborne pathogen in broiler production: Pathophysiology and potential strategies for controlling necrotic enteritis. Animals.

[36] Savva C.G., Clark A.R., Naylor C.E., Popoff M.R., Moss D.S., Basak A.K., Titball R.W., Bokori-Brown M. (2019). The pore structure of Clostridium perfringens epsilon toxin. Nat. Commun..

[37] Bruggisser J., Iacovache I., Musson S.C., Degiacomi M.T., Posthaus H., Zuber B. (2022). Cryo-EM structure of the octameric pore of Clostridium perfringens β-toxin. EMBO Rep..

[38] Ogbu C.P., Kapoor S., Vecchio A.J. (2023). Structural Basis of *Clostridium perfringens* Enterotoxin Activation and Oligomerization by Trypsin. Toxins.

[39] Yan X.X., Porter C.J., Hardy S.P., Steer D., Smith A.I., Quinsey N.S., Hughes V., Cheung J.K., Keyburn A.L., Kaldhusdal M. (2013). Structural and functional analysis of the pore-forming toxin NetB from Clostridium perfringens. mBio.

[40] Profeta F., Di Francesco C.E., Di Provvido A., Scacchia M., Alessiani A., Di Giannatale E., Marruchella G., Orsini M., Toscani T., Marsilio F. (2020). Prevalence of *netB*-positive *Clostridium perfringens* in Italian poultry flocks by environmental sampling. J. Vet. Diagn. Investig..

[41] Nagahama M., Takehara M., Seike S., Sakaguchi Y. (2023). Cellular Uptake and Cytotoxicity of *Clostridium perfringens* Iota-Toxin. Toxins.

[42] Yonogi S., Matsuda S., Kawai T., Yoda T., Harada T., Kumeda Y., Gotoh K., Hiyoshi H., Nakamura S., Kodama T. (2014). BEC, a novel enterotoxin of Clostridium perfringens found in human clinical isolates from acute gastroenteritis outbreaks. Infect. Immun..

[43] Takehara M., Takagishi T., Seike S., Oda M., Sakaguchi Y., Hisatsune J., Ochi S., Kobayashi K., Nagahama M. (2017). Cellular Entry of Clostridium perfringens Iota-Toxin and Clostridium botulinum C2 Toxin. Toxins.

[44] Schwan C., Lang A.E., Schlosser A., Fujita-Becker S., AlHaj A., Schröder R.R., Faix J., Aktories K., Mannherz H.G. (2022). Inhibition of Arp2/3 complex after ADP-ribosylation of Arp2 by binary Clostridioides toxins. Cells.

[45] Martínez-Meléndez A., Cruz-López F., Morfin-Otero R., Maldonado-Garza H.J., Garza-González E. (2022). An update on *Clostridioides difficile* binary toxin. Toxins.

[46] O’Grady K., Knight D.R., Riley T.V. (2021). Antimicrobial resistance in *Clostridioides difficile*. Eur. J. Clin. Microbiol. Infect. Dis..

[47] Czepiel J., Dróżdż M., Pituch H., Kuijper E.J., Perucki W., Mielimonka A., Goldman S., Wultańska D., Garlicki A., Biesiada G. (2019). Clostridium difficile infection: Review. Eur. J. Clin. Microbiol. Infect. Dis..

[48] Papatheodorou P., Carette J.E., Bell G.W., Schwan C., Guttenberg G., Brummelkamp T.R., Aktories K. (2011). Lipolysis-stimulated lipoprotein receptor (LSR) is the host receptor for the binary toxin Clostridium difficile transferase (CDT). Proc. Natl. Acad. Sci. USA.

[49] Alam M.Z., Madan R. (2024). *Clostridioides difficile* Toxins: Host Cell Interactions and Their Role in Disease Pathogenesis. Toxins.

[50] Wigelsworth D.J., Ruthel G., Schnell L., Herrlich P., Blonder J., Veenstra T.D., Carman R.J., Wilkins T.D., Van Nhieu G.T., Pauillac S. (2012). CD44 Promotes intoxication by the clostridial iota-family toxins. PLoS ONE.

[51] Nagahama M., Yamaguchi A., Hagiyama T., Ohkubo N., Kobayashi K., Sakurai J. (2004). Binding and internalization of Clostridium perfringens iota-toxin in lipid rafts. Infect. Immun..

[52] Hale M.L., Marvaud J.C., Popoff M.R., Stiles B.G. (2004). Detergent-resistant membrane microdomains facilitate Ib oligomer formation and biological activity of Clostridium perfringens iota-toxin. Infect. Immun..

[53] Blonder J., Hale M.L., Chan K.C., Yu L.R., Lucas D.A., Conrads T.P., Zhou M., Popoff M.R., Issaq H.J., Stiles B.G. (2005). Quantitative profiling of the detergent-resistant membrane proteome of iota-b toxin induced vero cells. J. Proteome Res..

[54] Papatheodorou P., Hornuss D., Nölke T., Hemmasi S., Castonguay J., Picchianti M., Aktories K. (2013). Clostridium difficile binary toxin CDT induces clustering of the lipolysis-stimulated lipoprotein receptor into lipid rafts. mBio.

[55] Landenberger M., Nieland J., Roeder M., Nørgaard K., Papatheodorou P., Ernst K., Barth H. (2021). The cytotoxic effect of *Clostridioides difficile* pore-forming toxin CDTb. Biochim. Biophys. Acta Biomembr..

[56] Nagahama M., Nagayasu K., Kobayashi K., Sakurai J. (2002). Binding component of Clostridium perfringens iota-toxin induces endocytosis in Vero cells. Infect. Immun..

[57] Knapp O., Benz R., Gibert M., Marvaud J.C., Popoff M.R. (2002). Interaction of Clostridium perfringens iota-toxin with lipid bilayer membranes. Demonstration of channel formation by the activated binding component Ib and channel block by the enzyme component Ia. J. Biol. Chem..

[58] Knapp O., Benz R., Popoff M.R. (2016). Pore-forming activity of clostridial binary toxins. Biochim. Biophys. Acta.

[59] Goldsmith J.A., Dewar V., Hermand P., Blais N., McLellan J.S. (2023). Structural Basis for Binding of Neutralizing Antibodies to. J. Bacteriol..

[60] Gibert M., Monier M.N., Ruez R., Hale M.L., Stiles B.G., Benmerah A., Johannes L., Lamaze C., Popoff M.R. (2011). Endocytosis and toxicity of clostridial binary toxins depend on a clathrin-independent pathway regulated by Rho-GDI. Cell. Microbiol..

[61] Machen A.J., Fisher M.T., Freudenthal B.D. (2021). Anthrax toxin translocation complex reveals insight into the lethal factor unfolding and refolding mechanism. Sci. Rep..

[62] Ernst K., Schnell L., Barth H. (2017). Host Cell Chaperones Hsp70/Hsp90 and Peptidyl-Prolyl Cis/Trans Isomerases Are Required for the Membrane Translocation of Bacterial ADP-Ribosylating Toxins. Curr. Top. Microbiol. Immunol..

[63] Ernst K., Schmid J., Beck M., Hägele M., Hohwieler M., Hauff P., Ückert A.K., Anastasia A., Fauler M., Jank T. (2017). Hsp70 facilitates trans-membrane transport of bacterial ADP-ribosylating toxins into the cytosol of mammalian cells. Sci. Rep..

[64] Haug G., Aktories K., Barth H. (2004). The host cell chaperone Hsp90 is necessary for cytotoxic action of the binary iota-like toxins. Infect. Immun..

[65] Ernst K., Sailer J., Braune M., Barth H. (2021). Intoxication of mammalian cells with binary clostridial enterotoxins is inhibited by the combination of pharmacological chaperone inhibitors. Naunyn Schmiedebergs Arch. Pharmacol..

[66] Ernst K. (2022). Requirement of Peptidyl-Prolyl Cis/Trans isomerases and chaperones for cellular uptake of bacterial AB-type toxins. Front. Cell. Infect. Microbiol..

[67] Ernst K., Liebscher M., Mathea S., Granzhan A., Schmid J., Popoff M.R., Ihmels H., Barth H., Schiene-Fischer C. (2016). A novel Hsp70 inhibitor prevents cell intoxication with the actin ADP-ribosylating Clostridium perfringens iota toxin. Sci. Rep..

[68] Miller C.J., Elliott J.L., Collier R.J. (1999). Anthrax protective antigen: Prepore-to-pore conversion. Biochemistry.

[69] Albrecht T., Zhao Y., Nguyen T.H., Campbell R.E., Johnson J.D. (2015). Fluorescent biosensors illuminate calcium levels within defined beta-cell endosome subpopulations. Cell Calcium.

[70] Abrami L., Lindsay M., Parton R.G., Leppla S.H., van der Goot F.G. (2004). Membrane insertion of anthrax protective antigen and cytoplasmic delivery of lethal factor occur at different stages of the endocytic pathway. J. Cell Biol..

